# Comparison of laser guidance and freehand hook-wire for CT-guided preoperative localization of pulmonary nodules

**DOI:** 10.1186/s13019-024-02706-x

**Published:** 2024-04-05

**Authors:** Zijian Li, Ziyue Zhou, Kunpeng Feng, Xinyu Song, Chun Xu, Chang Li, Jun Zhao, Li Ye, Ziqing Shen, Cheng Ding

**Affiliations:** 1 Department of Thoracic Surgery, The First Affiliated Hospital of Soochow University, Medical College of Soochow University, Suzhou, 215000 China; 2 Institute of Thoracic Surgery, The First Affiliated Hospital of Soochow University, Suzhou, China; 3 Department of Marketing, Neorad Medical Technology (Shanghai) Co., Ltd., Shanghai, 201100 China

**Keywords:** Hook-wire localization, Laser guidance technology, Uni-port video-assisted thoracic surgery (VATS)

## Abstract

**Purpose:**

In VATS surgery, precise preoperative localization is particularly crucial when dealing with small-diameter pulmonary nodules located deep within the lung parenchyma. The purpose of this study was to compare the efficacy and safety of laser guidance and freehand hook-wire for CT-guided preoperative localization of pulmonary nodules.

**Methods:**

This retrospective study was conducted on 164 patients who received either laser guidance or freehand hook-wire localization prior to Uni-port VATS from September 1st, 2022 to September 30th, 2023 at The First Affiliated Hospital of Soochow University. Patients were divided into laser guidance group and freehand group based on which technology was used. Preoperative localization data from all patients were compiled. The localization success and complication rates associated with the two groups were compared. The risk factors for common complications were analyzed.

**Results:**

The average time of the localization duration in the laser guidance group was shorter than the freehand group (*p*<0.001), and the average CT scan times in the laser guidance group was less than that in the freehand group (*p*<0.001). The hook-wire was closer to the nodule in the laser guidance group (*p*<0.001). After the localization of pulmonary nodules, a CT scan showed 14 cases of minor pneumothorax (22.58%) in the laser guidance group and 21 cases (20.59%) in the freehand group, indicating no statistical difference between the two groups (*p*=0.763). CT scans in the laser guidance group showed pulmonary minor hemorrhage in 8 cases (12.90%) and 6 cases (5.88%) in the freehand group, indicating no statistically significant difference between the two groups (*p*=0.119). Three patients (4.84%) in the laser guidance group and six patients (5.88%) in the freehand group had hook-wire dislodgement, showing no statistical difference between the two groups (*p*=0.776).

**Conclusion:**

The laser guidance localization method possessed a greater precision and less localization duration and CT scan times compared to the freehand method. However, laser guidance group and freehand group do not differ in the appearance of complications such as pulmonary hemorrhage, pneumothorax and hook-wire dislodgement.

## Background

In recent years, lung cancer has ranked among the highest incidence and mortality rates for malignant tumors globally [1]. The radiological manifestations of early-stage lung cancer often appear in the forms of pulmonary nodules. With the widespread use of computed tomography (CT), the detection rate of pulmonary nodules has gradually increased. The definite determination of the benign or malignant nature of pulmonary nodules relies on pathological examination.

Video-assisted thoracoscopic surgery (VATS) was introduced into clinical practice in the 1990s and has now surpassed traditional open chest surgery, becoming the preferred approach for treating pulmonary nodules [2]. During VATS procedures, for solid nodules adjacent to the pleura, surgeons can determine the nodule’s position through visual observation of pleural depression and involvement, tactile sensation with fingers, and instrument sliding. This helps in selecting an appropriate resection range. However, for the majority of pulmonary nodules that are far from the pleura, the methods mentioned above cannot accurately locate their specific positions. To achieve precise resection, ensure sufficient and safe margins, avoid unnecessary enlargement of the resection area, preserve functional lung tissue as much as possible, and prevent medical dilemmas, it’s proposed that a requirement for localization of uncertain pulmonary nodules should be established.

Currently, hook-wire localization is the most widely used preoperative localization method, known for its simplicity, speed, high accuracy, and relatively low cost. Researches indicate that laser guidance methods have higher precision compared to the freehand approach [3]. In biopsy and ablation surgery, incorporating laser guidance can significantly reduce fluoroscopy time [4, 5].

The objective of this study is to compare the efficacy of hook-wire localization for pulmonary nodules under laser guidance and freehand CT-guided methods, as well as to compare the difference of incidence rate of common complications of both methods.

## Method

### Study population

**Fig. 1:**
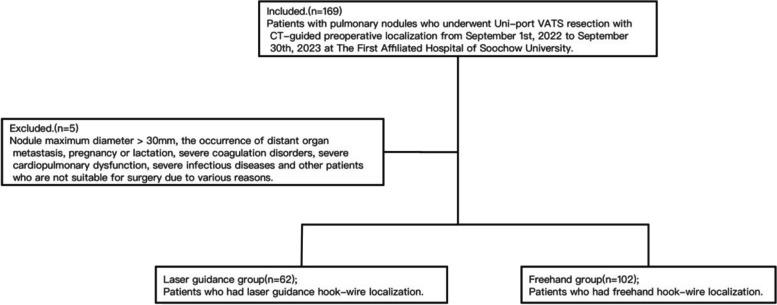
Included: Patients with pulmonary nodules who underwent Uni-port VATS resection with CT-guided preoperative localization from September 1st, 2022 to September 30th, 2023 at The First Affiliated Hospital of Soochow University. Excluded: Nodule maximum diameter>30mm, the occurrence of distant organ metastasis, pregnancy or lactation, severe coagulation disorders, severe cardiopulmonary dysfunction, severe infectious diseases and other patients who are not suitable for surgery due to various reasons

A retrospective analysis was performed for 164 patients with pulmonary nodules who underwent Uni-port VATS resection with CT-guided preoperative localization from September 1st, 2022 to September 30th, 2023 at The First Affiliated Hospital of Soochow University. All patients received CT-guided hook-wire preoperative localization. Among them, 62 patients had laser guidance hook-wire localization and 102 patients had freehand hook-wire localization. After preoperative localization, both groups had Uni-port VATS anatomic segmentectomy or wedge resection. Prior to the surgery, each case underwent comprehensive preoperative preparation and discussion. All patients engaged in detailed preoperative discussions with clinical doctors and signed written informed consent. This study obtained approval from the ethical review board from The First Affiliated Hospital of Soochow University. Inclusion criteria are as follows. Chest CT imaging confirmed the presence of pulmonary nodules (diameter≤30mm) without distant metastasis. CT findings of the pulmonary nodule indicated a high likelihood of malignancy. The lesion was located in the peripheral region of the lung, making it difficult to locate during surgery. The coagulation function is normal. Exclusion criteria included: Nodule maximum diameter>30mm, the occurrence of distant organ metastasis, pregnancy or lactation, severe coagulation disorders, severe cardiopulmonary dysfunction, severe infectious diseases and other patients who are not suitable for surgery due to various reasons (Fig. 1).

### Preoperative localization

Main materials: hook-wire needle (18G*10cm, GALLINIS.R.L, Italy). Patients were transferred to the CT room 2 hours before surgery. The optimal positioning, including supine, prone, or lateral decubitus, was determined based on the pre-admission chest CT scan showing the nodule's location. Radiopaque markers were placed on the skin surface overlying the nodule. Chest CT scanning was performed, and, following the "nearest vertical" principle, the optimal puncture point, angle, and depth were planned, with a focus on avoiding deep muscles, intercostal arteries, pulmonary arteries, pulmonary veins, and bronchi as much as possible.

Using the CT laser-guided lines and in combination with the skin markers, a local sterilization was conducted at the skin marker puncture point. Aseptic draping was applied, and local anesthesia was administered with lidocaine. The hook-wire needle was inserted along the planned puncture path to the predetermined depth. A repeat CT scan was performed to confirm the hook-wire’s position and depth. Adjustments to the direction and depth were made as necessary, with the hook-wire positioned within 20mm of the nodule and ideally 15mm-20mm below the pleura. The anchor hook was released, and the puncture point was disinfected and covered with gauze. Another CT scan was performed to assess for complications such as dislocation, pneumothorax, pulmonary bleeding, or hemothorax.

In the freehand group, the first needle insertion was made approximately 2-3cm deep into the patient's chest wall, ensuring that the puncture did not breach the parietal pleura. Radiologists then adjusted the CT scan range and performed a second CT scan. Based on the CT images displaying the position of the needle relative to the pulmonary nodule, adjustments were made to the needle's insertion path and depth. After releasing the hook-wire, another CT scan was performed to assess the presence of complications. This method involved manual adjustments guided by real-time CT imaging and was performed under the supervision of radiologists.

**Fig. 2:**
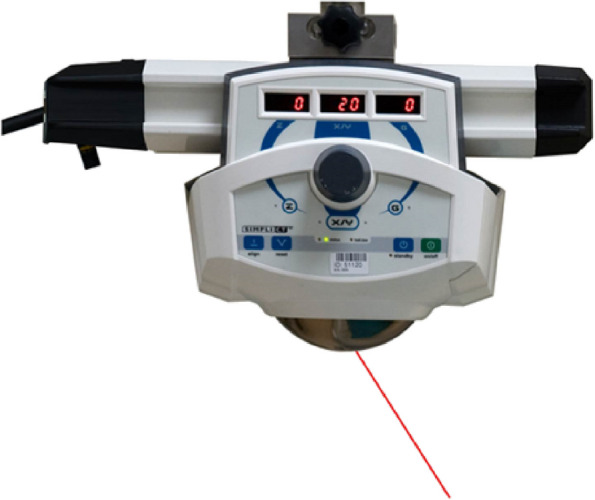
SimpliCT device

**Fig. 3:**
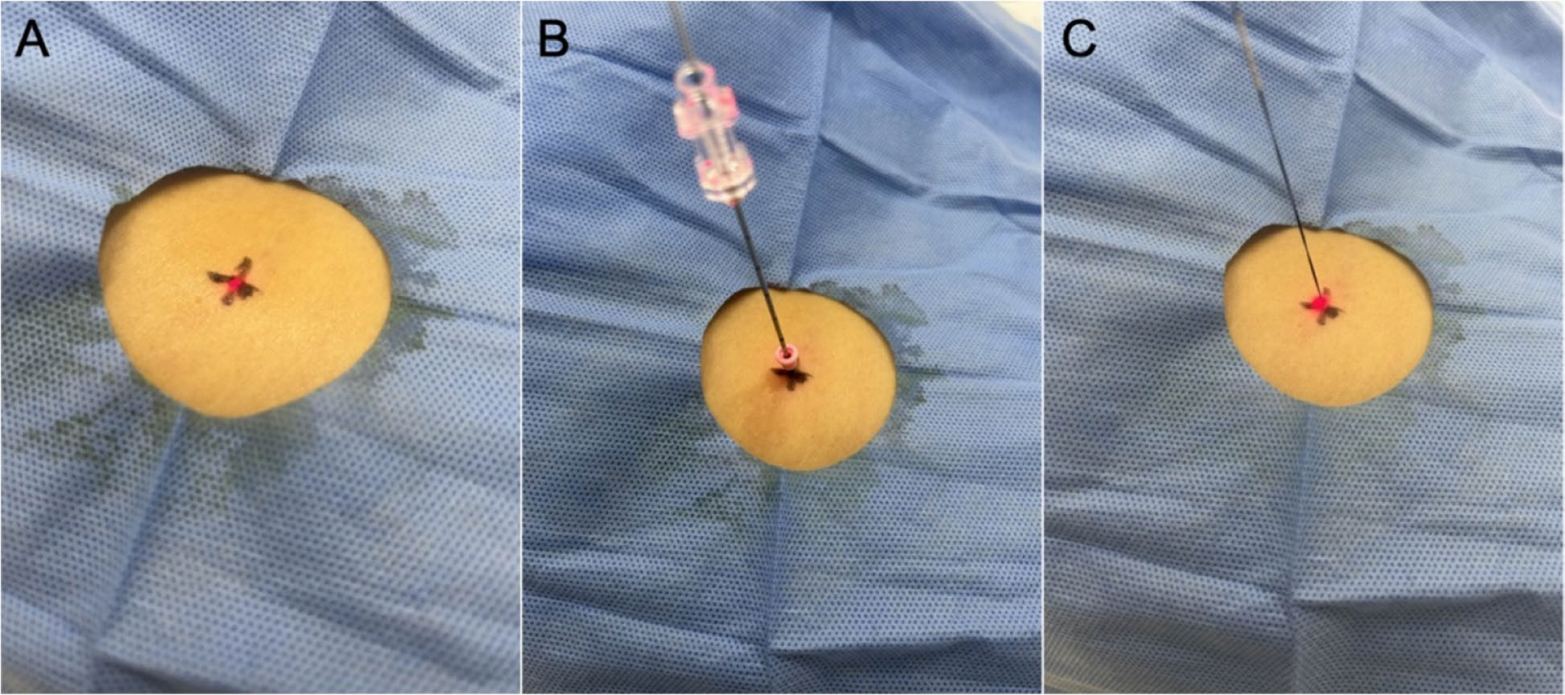
The localization process of laser guidance group. **a** After identifying the surface puncture point, adjust the laser machine accordingly. **b** Puncture along the guidance path of the laser. **c** After completing the puncture, release the sheath of the puncture needle and retain the hook-wire

**Fig. 4:**
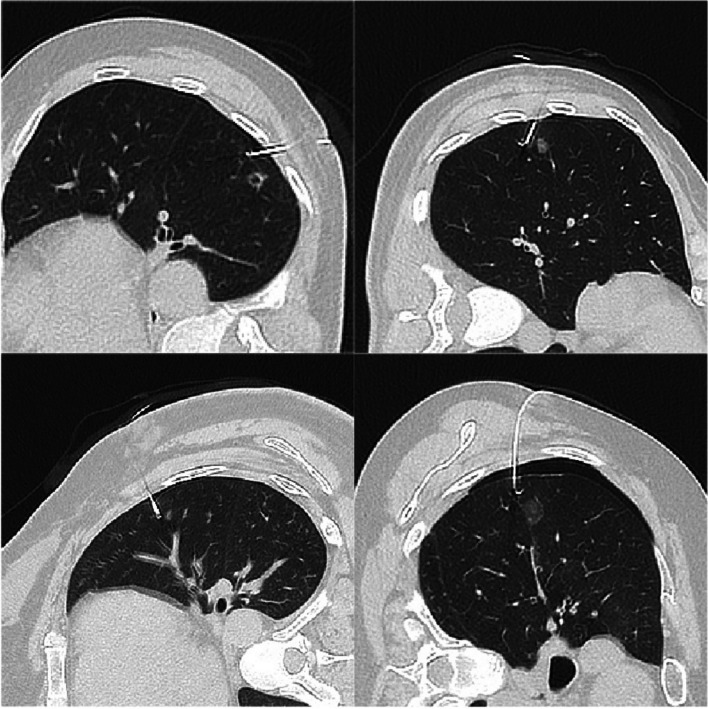
CT scanning was performed after the localization to confirm the accuracy

SimpliCT (NeoRad AS, Oslo, Norway) is a laser-based guidance device for CT-guided percutaneous interventions (Fig. 2). The laser guidance acts as a laser pointing device to visualize the planned needle path (possible to 45° in the transversal and sagittal planes) for the operator. In the laser guidance group, the operator used the laser pathway provided by SimpliCT for guidance during the puncture. After inserting the needle to the appropriate depth, the hook-wire was released. During this procedure, CT scanning was performed periodically to confirm the accuracy of the localization (Figs. 3 and 4).

### Working principle of SimpliCT

**Fig. 5:**
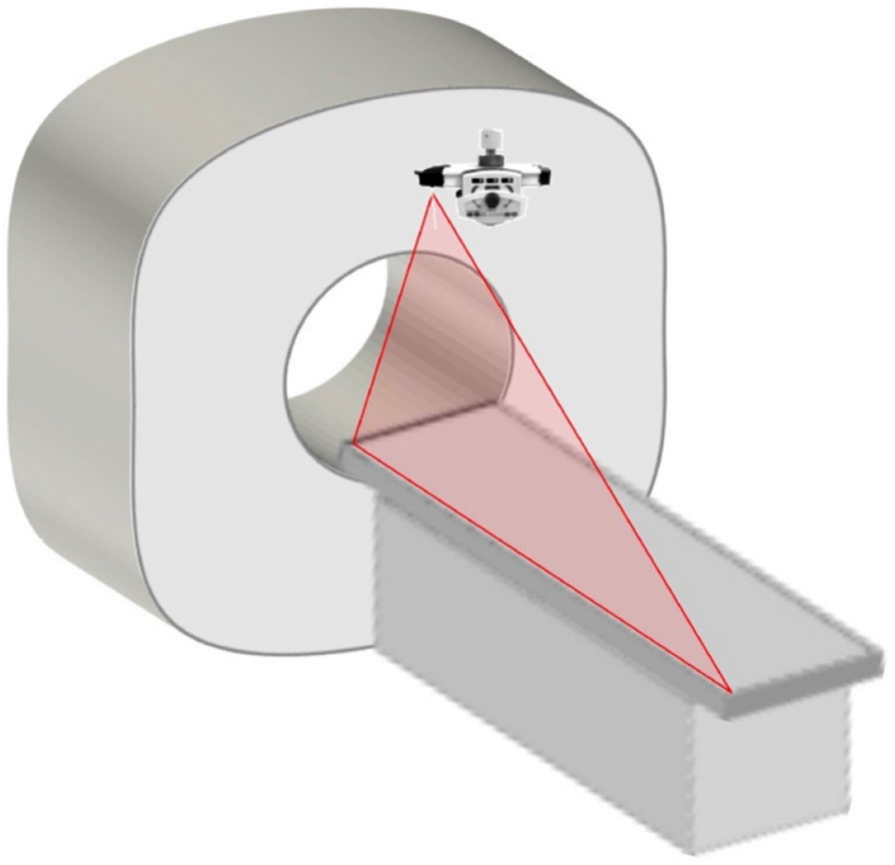
The Line Laser provides a line that is used for aligning SimpliCT

**Fig. 6:**
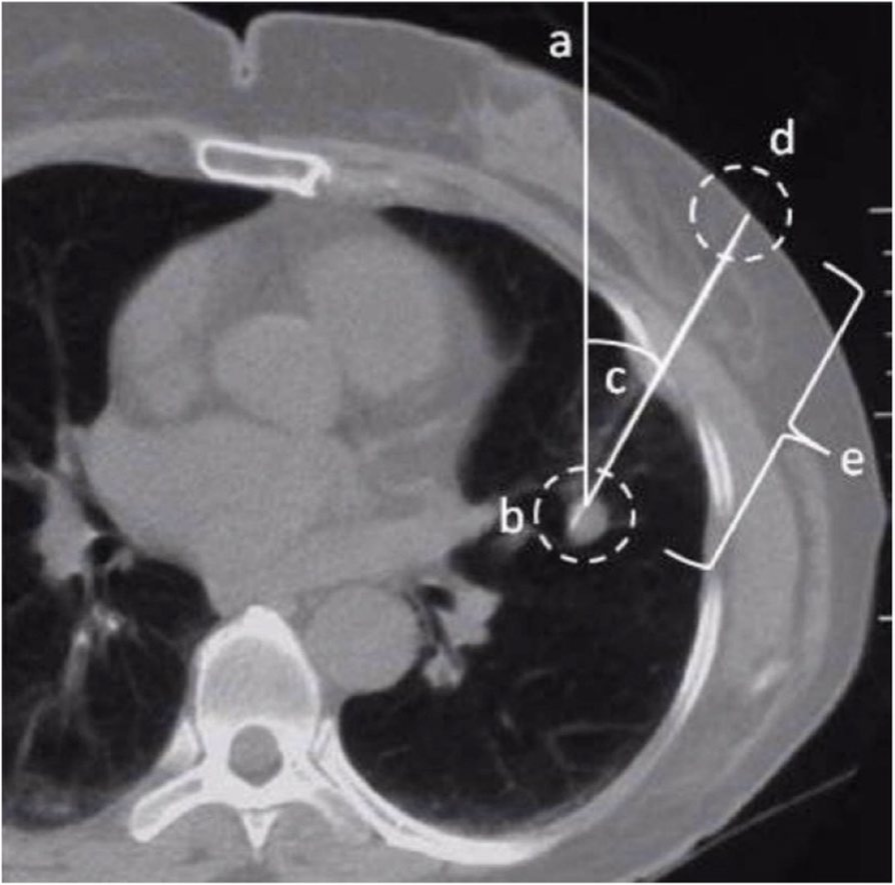
In the image a cross section of a human thorax is shown. **a** True vertical of the CT coordinate system, **b** target, **c** angle with respect to the vertical line, **d** entry point, **e** depth

Before the puncture, the Line Laser in the SimpliCT device need to be turned on to provide a line that is used for aligning SimpliCT, parallel to the CT table, as is shown in Fig. 5. The Line Laser can be used with the Laser Unit in either side of the CT table. After that, the patient is placed on the CT table in a given situation and a CT scan is taken. In the image of the first CT scan, the entry point and the angle with respect to the vertical line, together with the depth of puncture can be measured as is shown in Fig. 6. Set the angle with respect to the vertical line as is measured in the CT image in the SimpliCT device and adjust the position of the CT table and the SimpliCT device. Make sure that the end of the laser point meets with the entry point. The path guided by the laser beam is the best puncture path.

The laser device, also known as SimpliCT, contains Class 2 lasers (IEC 60825-1:2014). The user and the patient should avoid staring into the laser beam. In addition, the CE mark confirms that SimpliCT complies with the Medical Device Directive (93/42/EEC).

### During surgery

After the localization was completed, all patients were immediately transported to the operating room. During surgery, the positioning needle was visually confirmed to be fixed to the pleural surface, indicating successful localization. The area containing the nodule was identified based on the position of the localization needle on the pleura and the traction of the tail thread, and a wedge resection was performed. The surgical approach involved performing the wedge resection first, ensuring a margin of at least 20mm from the nodule. After resection, the specimen was sent for rapid frozen section pathology examination. If the rapid pathology results indicated invasive adenocarcinoma and the patient's lung function allowed, a lobectomy and mediastinal lymph node dissection were performed.

### Statistical analysis

Descriptive statistics for continuous variables are presented as mean ± SD. Continuous variables were analyzed using Student’s t-test. However, categorical variables were compared using the Chi-square test or Fisher’s exact test. P values less than 0.05 were considered statistically significant. All statistical analyses were performed using spss26.0 software.

## Results

A total of 62 patients were included in the laser guidance group, including 19 males and 43 females. The average age was 53±13.3 years(range: 28-82 years). The average BMI(body mass index) was 23.4±2.8 kg/m^2^(range:17.97-31.22 kg/m^2^). The average distance between the pulmonary nodules and the pleural surface was 8.16±6.54mm(range:0-23mm). There were 102 patients in the freehand group, including 39 males and 63 females. The average age was 53.2±11.5 years(range:24-77 years). The average BMI(body mass index) was 24.0±3.4 kg/m^2^(range:18.03-32.05 kg/m^2^). The average distance between the pulmonary nodules and the pleural surface was 6.44±5.87mm(range:0-21mm). The factors mentioned above did not vary between the two patient groups significantly. The average CT nodule size was 10.31±3.33mm(range:4-23mm) in the laser guidance group and the average CT nodule size was 9.42±3.64mm(range:4-18mm) in the freehand group. There was no statistical difference between the two groups (*p*=0.084).

Within the laser guidance group, localization was in the right upper lobe in 24 patients (38.71%), in the middle lobe in 4 patients (6.45%), in the right lower lobe in 13 patients (20.97%), in the left upper lobe in 14 patients (22.58%), and in the left lower lobe in 7 patients (11.29%). In the freehand group, 36 patients (35.29%) showed localization in the right upper lobe, 6 patients (5.88%) in the middle lobe, 22 patients (21.57%) in the right lower lobe, 21 patients (20.59%) in the left upper lobe, and 17 patients (16.67%) in the left lower lobe. There was no statistical difference between the two groups (*p*=0.912).

In the laser guidance group, 46 patients (74.19%) were put in the lateral position, 6 patients (9.68%) were put in the prone position and 10 patients (16.13%) were put in the supine position. Meanwhile, in the freehand group, 84 patients (82.35%) were put in the lateral position, 12 patients (11.76%) were put in the prone position and 6 patients (5.88%) were put in the supine position. There was no statistical difference between the two groups (*p*=0.099).

Pathological examination of the laser guidance group showed 7 cases (11.29%) of chronic inflammation, 9 cases (14.52%) of adenocarcinoma in situ, 35 cases (56.45%) of minimally invasive adenocarcinoma, and 11 cases (17.74%) of invasive adenocarcinoma. Within the freehand group, pathological examination showed 15 cases (14.71%) of chronic inflammation, 9 cases (8.82%) of adenocarcinoma in situ, 45 cases (44.12%) of minimally invasive adenocarcinoma, and 33 cases (32.35%) of invasive adenocarcinoma. There was no statistical difference between the two groups (*p*=0.125).

In the laser guidance group, 4 patients (6.45%) underwent anatomic lobectomy, 5 patients (8.06%) underwent anatomic segmentectomy and 53 patients (85.48%) underwent wedge resection. In the freehand group, 6 patients (5.88%) underwent anatomic lobectomy, 12 patients (11.76%) underwent anatomic segmentectomy, and 84 patients (82.35%) underwent wedge resection. There was no statistical difference between the two groups (*p*=0.750). All nodules were successfully localized, and Uni-port VATS resection was completed in all the patients.

After the localization of pulmonary nodules, a CT scan showed 14 cases of minor pneumothorax (22.58%) in the laser guidance group and 21 cases (20.59%) in the freehand group, indicating no statistical difference between the two groups (*p*=0.763). Although both groups of patients had minor pneumothorax, they had no obvious symptoms of chest tightness or discomfort, and no chest tube insertion was required for drainage. CT scans in the laser guidance group showed pulmonary minor hemorrhage in 8 cases (12.90%) and 6 cases (5.88%) in the freehand group, indicating no statistically significant difference between the two groups (*p*=0.119). However, neither group of patients showed any serious clinical consequences. Moreover, no hemothorax or pleural reaction was reported in either group of patients. Three patients (4.84%) in the laser guidance group and six patients (5.88%) in the freehand group had hook-wire dislodgement, showing no statistical difference between the two groups (*p*=0.776) (Table 1).

**Table 1:** Comparison of Variables between the 2 groups

	Laser guidance group	Freehand group	*P* value
Gender (Male/Female)	19/43	39/63	0.324
Age (years)	53.0±13.3	53.2±11.5	0.944
BMI (kg/m^2^)	23.4±2.8	24.0±3.4	0.197
**Nodule location**			0.912
RUL	24(38.71%)	36(35.29%)	
LUL	14(22.58%)	21(20.59%)	
RML	4(6.45%)	6(5.88%)	
RLL	13(20.97%)	22(21.57%)	
LLL	7(11.29%)	17(16.67%)	
CT nodule size(mm)	10.31±3.33	9.42±3.64	0.084
**Position**			0.099
lateral	46(74.19%)	84(82.35%)	
prone	6(9.68%)	12(11.76%)	
supine	10(16.13%)	6(5.88%)	
Nodule distance to pleural surface(mm)	8.16±6.54	6.44±5.87	0.083
Localization duration(min)	8.91±1.43	12.77±1.94	<0.001**
CT scan times	3.67±0.67	4.05±0.59	<0.001**
Localization depth within the lung parenchyma(mm)	13.17±3.6	15.64±5.62	0.002*
The accuracy of localization(mm)	6.46±5.86	13.76±9.90	<0.001**
Mild pulmonary hemorrhage			0.119
Yes	8	6	
No	54	96	
Minor pneumothorax			0.763
Yes	0	0	
No	62	102	
Pleural reaction			
Yes	0	0	
No	62	102	
Hook-wire dislodgement			0.776
Yes	3	6	
No	59	96	
Operation time of wedge resection (min)	17.83±4.64	19.35±6.97	0.131
Operation time(min)	80.03±43.68	75.20±48.53	0.523
**Surgical type**			0.750
Wedge resection	53(85.48%)	84(82.35%)	
Segmentectomy	5(8.06%)	12(11.76%)	
Lobectomy	4(6.45%)	6(5.88%)	
**Histopathologic results**			0.125
Benign lesion	7(11.29%)	15(14.71%)	
AIS	9(14.52%)	9(8.82%)	
MIA	35(56.45%)	45(44.12%)	
IA	11(17.74%)	33(32.35%)	

*BMI* body mass index, *RUL* right upper lobe, *LUL* left upper lobe, *RML* right middle lobe, *RLL* right lower lobe, *LLL* left lower lobe, *CT* computed tomography, *AIS* adenocarcinoma in situ, *MIA* minimally invasive adenocarcinoma, *IA* invasive adenocarcinoma

^*^p<0.01, **p<0.001 (student’s t-test; chi-squared test)

During the operation, the operation time of wedge resection in the laser guidance group was 17.83±4.64min and in the freehand group was 19.35±6.97min, indicating no statistical difference between the two groups (*p*=0.131). Moreover, the overall Uni-port VATS operation time was 80.03±43.68min in the laser guidance group and 75.20±48.53min in the freehand group, indicating no statistical difference between the two groups (*p*=0.523).

The average time from the start of the first CT scan to the end of the last CT scan in the laser guidance group was 8.91±1.43min (range:6.5-13.5min), and the average time from the start of the first CT scan to the end of the last CT scan in the freehand group was 12.77±1.94min (range:8.0-16.0min), indicating a statistical difference between the two groups (*p*<0.001). During the localization, the average CT scan times in the laser guidance group was 3.67±0.67 times (range:3-5 times), and the average CT scan times in the freehand group was 4.05±0.59 times (range:3-6 times), indicating a statistical difference between the two groups (*p*<0.001). In the CT image of the localization, the accuracy of localization, quantified as the distance between the hook-wire and the nodule in the lung surface, was 6.46±5.86mm (range:0-30mm) in the laser guidance group, and 13.76±9.90mm (range:0-44mm), indicating a statistical difference between the two groups (*p*<0.001).

## Discussion

In recent years, lung cancer has ranked among the most prevalent and deadliest malignancies globally [1]. In China, there has been an increasing emphasis on health check-ups. This has led to a rising detection rate of pulmonary nodules. Before the surgical resection of pulmonary nodules, a safe and precise preoperative localization is badly needed. Meanwhile, the laser guidance technology is a new technology and performs a role of making the puncture operation more precise [3]. As a result, this study combines the laser guidance technology and preoperative localization together to find out whether the laser guidance technology can contribute positively.

In recent years, Uni-port VATS techniques have gained popularity due to their advantages, including minimal surgical trauma and rapid postoperative recovery [6–8]. However, Uni-port VATS procedures involve smaller incisions, making it challenging for surgeons to accurately determine the location of pulmonary nodules through tactile feedback alone. Consequently, preoperative CT-guided precise localization techniques for pulmonary nodules have gradually matured. In the process, preoperative localization methods are divided into two main categories based on the different paths of localization: percutaneous lung localization and bronchoscopic localization, including hook-wire localization [9, 10], intraoperative ultrasound [11, 12], medical glue [13], micro-coil [14], methylene blue [15], indocyanine green [16], and radionuclides [17]. In the study of micro-coil localization, 3% patients had pneumothorax requiring chest tube placement but no patients in our study placed chest tube for any reason after the localization. Meanwhile, the medical glue and the indocyanine green method possess a similar possibility of occurrence of complications compared to the hook-wire method. One of the most widely used clinical localization techniques is the hook-wire localization method due to its relatively stable localization effect and cost-effectiveness [18]. This study aims to investigate the effectiveness and safety of laser-guided localization methods based on the hook-wire localization technique compared to the traditional freehand hook-wire localization method.

There is existing research that demonstrates the significant reduction in intraoperative fluoroscopy time, up to 50%, when using laser guidance for the percutaneous radiofrequency ablation of osteoid osteoma under C-arm cone-beam CT guidance [5]. This improvement can be attributed to the SimpliCT machine's ability to provide a clear puncture path using laser beams during localization and ablation procedures. The angle and direction of this puncture path can be flexibly adjusted according to the purpose of the procedure. Operators can use the laser-projected path as a guide to puncture at the most ideal angle as planned.

The results show that compared to the freehand CT-guided puncture approach, the laser guidance method not only effectively reduces the time required for puncture localization but also significantly improves the accuracy of the puncture. It provides precise guidance for the accurate removal of pulmonary nodules during surgery. However, whether using the freehand or laser guidance method, both are based on the preoperative localization of hook-wire puncture under CT guidance. Therefore, the incidence of complications such as bleeding and pneumothorax does not significantly differ between these two localization methods. This is because the hook-wire localization method uses a relatively thick puncture needle, and the procedure involves sharp maneuvers, making bleeding difficult to avoid. Pneumothorax is also challenging to prevent after the release of the hook-wire and the withdrawal of the puncture needle's sheath.

In addition to the hook-wire method, there are several other methods for preoperative localization of pulmonary nodules. One of the least painful methods for patients is the use of electromagnetic navigation bronchoscopy localization under general anesthesia [19, 20]. Patients undergoing electromagnetic navigation bronchoscopy localization typically experience minimal additional pain beyond the surgery itself. However, this method has several disadvantages, including prolonged anesthesia time and an increased risk associated with anesthesia. Additionally, it can be costly, which may make it less accessible for some patients.

A study also suggest the use of intraoperative ultrasound localization [12]. In this study, the ultrasonographic identification of pulmonary nodules were used in two patients and all succeeded. This method shares similarities with magnetic navigation-assisted localization, as it spares patients from the physical discomfort and psychological distress associated with invasive procedures. Complications like pulmonary bleeding and pneumothorax, which may result from invasive procedures, can also be avoided. However, the effectiveness of ultrasound examination for locating pulmonary nodules during surgery may not be optimal, possibly due to the challenges posed by the high air content in lung tissue.

Similar localization methods to hook-wire include micro-coil, indocyanine green, methylene blue, medical adhesive, and the injection of mixed liquids. These methods all require preoperative procedures guided by CT. Their common advantage is that CT-guided imaging can enhance the accuracy and success rate of localization, providing a clear and precise localization result. However, these localization methods also share common disadvantages, such as severe pain, pneumothorax, pulmonary bleeding, and puncture site bleeding that can occur in patients due to the localization procedure before surgery. While using a slender needle tip to inject liquid can minimize the likelihood of these complications, the potential for these complications still exists.

In this study, our primary focus was on comparing the advantages of laser guidance-assisted hook-wire placement with the freehand hook-wire placement method. However, we believe that the application of laser guidance methods extends beyond this specific context. Further research is needed to investigate whether laser guidance, in combination with other localization methods such as micro-coil, indocyanine green, methylene blue, medical adhesive, and the injection of mixed liquids, offers superior localization results compared to the freehand method.

In the field of thoracic surgery, the ablation treatment of pulmonary nodules and percutaneous lung biopsy of lung masses are widely performed diagnostic and therapeutic procedures. The potential application of laser guidance methods in the ablation of pulmonary nodules and the percutaneous biopsy of lung masses also warrants further exploration. In the process of using laser positioning machines, we observed that these machines may reduce the learning curve for clinical doctors in performing localization procedures, thereby shortening the time required for clinicians to learn localization operations. This observation, however, requires further research for confirmation.

## Conclusion

In conclusion, adding laser guidance to CT-guided preoperative localization for puncture alignment with the planned needle path can effectively reduce the CT scan times and localization duration. Meanwhile, the laser guidance method can also bring the localization more precision. Therefore, the laser guidance method is helpful to clinical practice, and both patients and doctors can benefit from it. However, laser guidance group and freehand group do not differ in the appearance of complications such as pulmonary hemorrhage, pneumothorax and hook-wire dislodgement. This could be because both localization methods are all based on hook-wire needle, which is the main cause of complications.

## Data Availability

No datasets were generated or analysed during the current study.

## Abbreviations

VATS: Video-assisted thoracic surgery
BMI: Body mass index
RUL: Right upper lobe
LUL: Left upper lobe
RML: Right middle lobe
RLL: Right lower lobe
LLL: Left lower lobe
CT: Computed tomography
AIS: Adenocarcinoma in situ
MIA: Minimally invasive adenocarcinoma
IA: Invasive adenocarcinoma
